# Use of Salivary Iodine Concentrations to Estimate the Iodine Status of Adults in Clinical Practice

**DOI:** 10.1093/jn/nxab303

**Published:** 2021-09-29

**Authors:** Bernadette L Dekker, Daan J Touw, Anouk N A van der Horst-Schrivers, Michel J Vos, Thera P Links, D A Janneke Dijck-Brouwer, Anneke C Muller Kobold

**Affiliations:** Internal Medicine, Department of Endocrinology, University Medical Center Groningen, University of Groningen, Groningen, The Netherlands; Department of Clinical Pharmacy and Pharmacology, University Medical Center Groningen, University of Groningen, Groningen, The Netherlands; Internal Medicine, Department of Endocrinology, University Medical Center Groningen, University of Groningen, Groningen, The Netherlands; Department of Internal Medicine, Division of Endocrinology, Maastricht University Medical Center, Maastricht, The Netherlands; Department of Laboratory Medicine, University Medical Center Groningen, University of Groningen, Groningen, The Netherlands; Internal Medicine, Department of Endocrinology, University Medical Center Groningen, University of Groningen, Groningen, The Netherlands; Department of Laboratory Medicine, University Medical Center Groningen, University of Groningen, Groningen, The Netherlands; Department of Laboratory Medicine, University Medical Center Groningen, University of Groningen, Groningen, The Netherlands

**Keywords:** salivary iodine, urinary iodine, salivary protein, salivary urea, iodine intake

## Abstract

**Background:**

Measurement of the 24-h urinary iodine concentration or urinary iodine excretion (UIE) is the gold standard to determine iodine status; however, this method is inconvenient. The use of salivary iodine could be a possible alternative since salivary glands express the sodium-iodine symporter.

**Objectives:**

We aimed to establish the correlation between the salivary iodine secretion and UIE, to evaluate the clinical applicability of the iodine saliva measurement.

**Methods:**

We collected 24-h urine and saliva samples from 40 participants ≥18 y: 20 healthy volunteers with no specific diet (group 1), 10 patients with differentiated thyroid cancer with a low dietary intake (<50 μg/d, group 2), and 10 patients with a high iodine status as the result of the use of amiodarone (group 3). Urinary and salivary iodine were measured using a validated inductively coupled plasma MS method. To correct for differences in water content, the salivary iodine concentration (SIC) was corrected for salivary protein and urea concentrations (SI/SP and SI/SU, respectively). The intra- and inter-individual CVs were calculated, and the Kruskal-Wallis test and Spearman's correlation were used.

**Results:**

The intra-individual CVs for SIC, SI/SP, and SI/SU were 63.8%, 37.7%, and 26.9%, respectively. The inter-individual CVs for SIC, SI/SP, and SI/SU were 77.5%, 41.6% and 47.0%, respectively. We found significant differences (*P* < 0.01) in urinary and salivary iodine concentrations between all groups [the 24-h UIE values were 176 μg/d (IQR, 96.1–213 μg/d), 26.0 μg/d (IQR, 22.0–37.0 μg/d), and 10.0*10^3^ μg/d (IQR, 7.57*10^3^–11.4*10^3^ μg/d) in groups 1–3, respectively; the SIC values were 136 μg/L (IQR, 86.3–308 μg/L), 71.5 μg/L (IQR, 29.5–94.5 μg/L), and 14.3*10^3^ μg/L (IQR, 10.6*10^3^–25.6*10^3^ μg/L) in groups 1–3, respectively]. Correlations between the 24-h UIE and SIC, SI/SP, and SI/SU values were strong (ρ = 0.80, ρ = 0.90, and ρ = 0.86, respectively; *P* < 0.01).

**Conclusions:**

Strong correlations were found between salivary and urinary iodine in adults with different daily iodine intakes. A salivary iodine measurement can be performed to assess the total iodine body pool, with the recommendation to correct for salivary protein or urea.

## Introduction

Adequate iodine intake is required to synthesize thyroid hormones, with a recommended daily intake of 150 μg for adults (1, 2). To determine the iodine status, measurement of the 24-h urinary iodine concentration or urinary iodine excretion (UIE) is the gold standard. However, this procedure is logistically challenging, time consuming, and cumbersome. So, in clinical practice, a more manageable way to measure the iodine body pool would be of benefit (3). A possible solution was shown by Kim et al. (4), with the urinary-iodine-to-creatinine ratio (UI/Cr) from spot urine used to estimate the 24-h UIE. However, UIE shows a circadian rhythm, with the lowest value in the morning, which could hamper the interpretation of data. Saliva could be an alternative for urine since the salivary glands express the sodium-iodine symporter, resulting in iodine transport from the bloodstream (5–8). In addition, several other transporters [cystic fibrosis transmembrane conductance regulator, anoctamin 1, and PENDRIN (PDS protein, *SLC26A4 gene*)] are involved in secreting iodine in the salivary fluid (9). Compared to 24-h urine, saliva collection is a more sanitary, simple, and quick procedure. This method could be helpful in patients with differentiated thyroid carcinoma (DTC) to determine their iodine status before they receive radioactive iodine (^131^I) therapy. A substantial portion of DTC patients receive this therapy after a total thyroidectomy to destroy residual thyroid (cancer) tissue (10). To increase the uptake of ^131^I therapy, a low iodine store in the body is pursued and achieved with a low-iodine diet (LID; iodine intake < 50.0 μg/d) (3). The iodine status of these DTC patients represents compliance with the diet. In some cases, DTC patients cannot receive ^131^I therapy directly because of the recent use of amiodarone or iodinated contrast for diagnostic imaging (10). Both lead to an excessive amount of iodine, so in these patients, practical and quick measurement of the iodine body pool can guide physicians to plan the ^131^I therapy optimally.

For these reasons, we aimed to establish the correlation between the salivary iodine secretion and 24-h UIE to evaluate whether an iodine saliva measurement reflects the total iodine body pool in clinical practice. In addition, for clinical practice, we determined the variation in salivary iodine secretion during the day.

## Methods

### Study design and population

**FIGURE 1:**
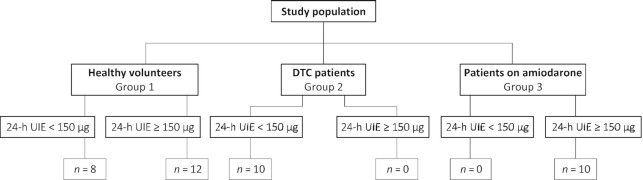
Overview of the study population. Group 1 consisted of 20 healthy volunteers with no specific diet, group 2 consisted of 10 DTC patients with low dietary iodine intake as part of their preparation for ^131^I therapy, and group 3 consisted of 10 patients using amiodarone. Abbreviations: ^131^I, radioactive iodine; 24-h UIE, urine iodine excretion per 24 h; DTC, differentiated thyroid carcinoma.

In this single-center pilot study performed at the University Medical Centre Groningen (UMCG), 40 participants ≥18 y with different amounts of daily iodine intake were included: 20 healthy volunteers with no specific diet (group 1); 10 DTC patients with a low dietary iodine intake (group 2) as part of their preparation for ^131^I therapy; and 10 patients with a high iodine status as the result of the use of amiodarone for more than 3 months (median dose of 200 mg/d; IQR, 200–225 mg/d; group 3; **Figure 1**). The exclusion criteria for this study were external radiotherapy of head and neck region in the medical history and pregnancy. The DTC patients and healthy volunteers did not use iodine-containing medication or supplements. Approval of the study by the Medical Ethics Committee of the UMCG was waived because this study was not assessed as clinical research with human subjects, as meant in the Medical Research Involving Human Subjects Act. No further Institutional Review Board approval was required. All participants gave written informed consent, and analyses were performed on fully anonymized data sets.

### Study definitions

To evaluate their iodine intake, the healthy volunteers were asked to note their food intake in a nutrition diary. According to the Dutch DTC guideline, the DTC patients were instructed to have an iodine intake of <50.0 μg/d for 7 d before receiving ^131^I therapy as part of their treatment (11). These patients also noted their food intake. The dietary intake of iodine of both the healthy volunteers and DTC patients was evaluated by 2 dietitians (LS-B and YV). According to the WHO, we defined a daily urinary iodine concentrations or excretions <150 μg/24-h as insufficient and those ≥150 μg/24-h as sufficient (sufficient iodine 24-h UIE: 100 μg/L*1.50 L) (12).

### Study method

The healthy volunteers collected *1*) urine in 2-h batches (8 subcollections) during the day (beginning at 07:00 or 08:00 and ending at 23:00 or 24:00) and 1 batch (1 subcollection) overnight (total of 24 h of urine collection) and *2*) saliva at the beginning of each subcollection of urine. The DTC patients collected *1*) 24 h of urine and *2*) 1 sample of morning saliva on day 7 of their LID. Patients on amiodarone collected *1*) 24 h of urine and *2*) morning saliva on the same day only once.

The collected urine was stored at -20°C until processing. Saliva was collected using a polyethylene swab (Salivettes, Sarstedt). Participants had to gently suck, without chewing, for 2–3 min on this Salivette. After collection, the saliva samples were centrifuged for 2 min at a relative centrifugal force of 1821 *g* and stored at −20°C until processing.

Participants were instructed to collect saliva without chewing because chewing leads to a higher water content of the saliva (stimulation parotid gland) (13, 14). However, since chewing cannot be excluded entirely, we decided to correct for the salivary water content using salivary protein and urea secretion. Salivary protein and urea are also diluted if the parotid gland is stimulated. Therefore, both salivary protein and urea were used to correct for dilution.

### Analytical methods

The urine and saliva iodine concentrations were measured using an inductively coupled plasma MS (Thermo Scientific iCap-TQ). Total inter- and intra-assay CV values were 2.1% to 2.9% at 40.0 μg/L, respectively, and 2.2% to 3.5% at 225 μg/L, respectively. The limit of quantification was 10.0 μg/L. Additional information about the urinary and salivary iodine measurements is available as **Supplemental Data**.

Urinary creatinine was analyzed using the enzymatic creatinine assay on the Roche Cobas C System.

Total saliva protein was analyzed after alkaline protein denaturation and addition of Benzethonium chloride by a turbidometric assay on the Roche Cobas Chemistry analyzer. Typical inter- and intra-assay CV values are 1.0%–2.0% over the entire measuring range. The limit of detection (LoD) was 40.0 mg/L.

The saliva urea concentration was measured using a kinetic test with urease and glutamate dehydrogenase assay on the Roche Cobas Chemistry analyzer. Typical inter- and intra-assay CV values are 0.9%–1.1% over the entire measuring range. The LoD was 0.50 mmol/L.

The UIE was expressed as daily urine iodine excretion (μg/d) based on 24-h urine samples and the UI/Cr (μg/gCr). Subjects with a total 24-h urine volume <600 mL were excluded from the 24-h UIE analysis.

To correct for differences in water content due to possible stimulation of salivary glands, the salivary iodine concentration (SIC) was not only expressed in μg/L but also in relation to the salivary protein and urea secretions [SI/SP (μg/g) and SI/SU (μg/mmol), respectively].

### Statistics

Data of urinary and/or salivary iodine, protein, and urea secretion of healthy volunteers during the day were expressed as means with SEMs or medians with IQRs. The intra- and inter-individual CVs were calculated according to Meijers et al. (15), with and without exclusion of the first sampling moment, since Guo et al. (16) observed a low correlation between SIC before breakfast and the previous day's iodine intake. The differences in the self-reported daily iodine intake and the salivary iodine concentrations of the healthy volunteers, subdivided based on their 24-h UIE values, were compared using the Mann-Whitney U test. The differences in the saliva secretion and urine excretion between the participants were compared with a Kruskal-Wallis test. In addition, Spearman's correlation was used to establish the correlations between the saliva secretion (SIC, SI/SP, and SI/SU) and urine excretion (24-h UIE and UI/Cr). *P* values <0.05 were considered significant. IBM SPSS Statistics for Windows, version 23.0 (IBM Corp) was used for the statistical analysis of the data.

## Results

### Participant characteristics

The median age of the healthy volunteers (*n* = 20; group 1) was 43.5 y (IQR 29.8–53.8), the median age of the DTC patients (*n* = 10; group 2) was 50.1 y (IQR, 44.0–59.0), and the median age of the patients on amiodarone (*n* = 10; group 3) was 71.5 y (IQR, 51.8–75.3). There were 11, 6, and 3 healthy volunteers, DTC patients, and patients on amiodarone who were female, respectively. The total 24-h urine volume of 1 DTC patient was <600 mL.

The healthy volunteers had a median iodine intake of 184 μg/d (IQR, 133–244 μg/d), and the median iodine intake of the DTC patients during the LID was 43.5 μg/d (IQR, 21.3–57.0 μg/d).

### Variation of salivary iodine in healthy volunteers

**FIGURE 2:**
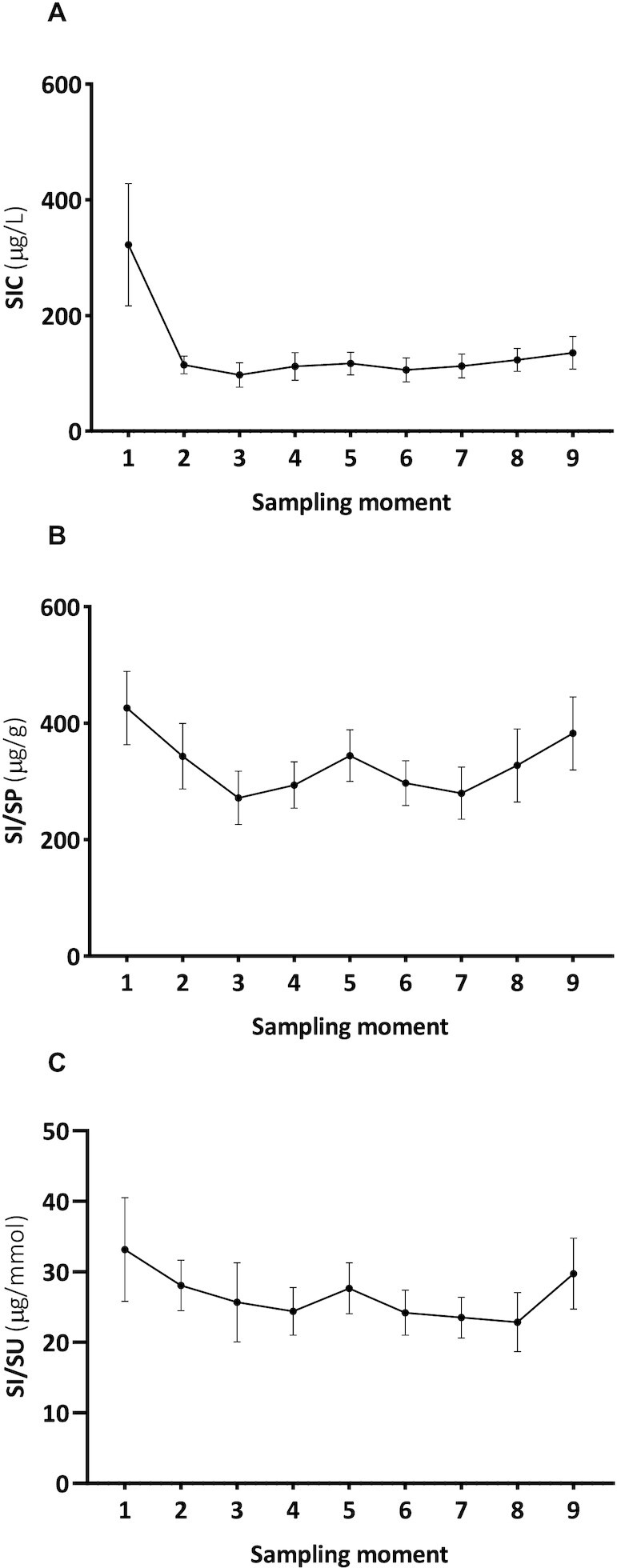
The mean (A) SIC, (B) SI/SP, and (C) SI/SU of 20 healthy volunteers (group 1) at different time points during the day. Twenty healthy volunteers collected 9 saliva samples over intervals of 2 h starting at 07:00 or 08:00 (sampling moment 1) and ending at 23:00 or 24:00 (sampling moment 9). Data are expressed as means (SEMs). Abbreviations: SIC, saliva iodine concentration; SI/SP, salivary-iodine-to-protein-ratio; SI/SU, salivary-iodine-to-urea ratio.

**Figure 2** represents the mean values of SIC, SI/SP, and SI/SU at different time points during the day. The intra-individual CVs for SIC, SI/SP, and SI/SU were 63.8%, 37.7%, and 26.9%, respectively. In addition, the inter-individual CVs for SIC, SI/SP, and SI/SU were 77.5%, 41.6%, and 47.0%, respectively. By excluding the first SIC sampling moment, an intra-individual CV of 39.8% and an inter-individual CV of 68.7% were found for SIC. The intra- and inter-individual CVs for SI/SP and SI/SU remained similar when the first sampling moment was excluded.

**FIGURE 3:**
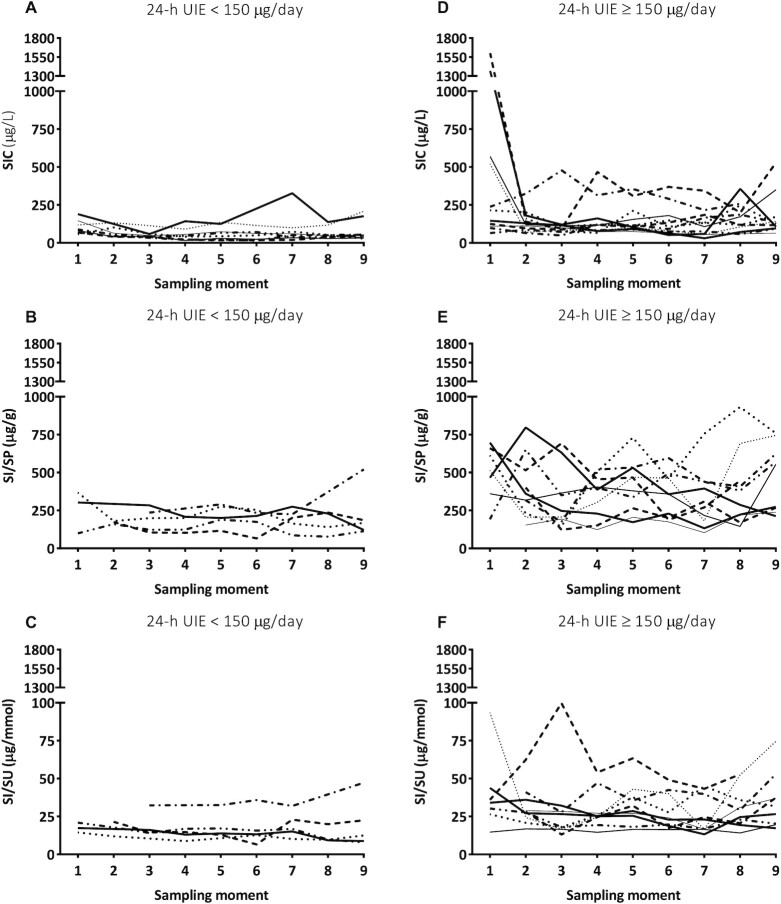
Individual SIC, SI/SP, and SI/SU data of 20 healthy volunteers (group 1) at different time points during the day with a 24-h UIE of (A–C) <150 μg/d (*n* = 8) or (D–F) ≥150 μg/d (*n* = 12). Twenty healthy volunteers collected 9 saliva samples at intervals of 2 h starting at 07:00 or 08:00 (sampling moment 1) and ending at 23:00 or 24:00 (sampling moment 9). Abbreviations: 24-h UIE, urine iodine excretion per 24 h, SIC, saliva iodine concentration; SI/SP, salivary-iodine-to-protein-ratio; SI/SU, salivary-iodine-to-urea ratio.

**Figure 3** represents the individual values of SIC, SI/SP, and SI/SU at different time points during the day. Healthy volunteers were subdivided based on their 24-h UIE (UIE < 150 μg/d compared with UIE ≥ 150 μg/d). The median self-reported daily iodine intake of the healthy volunteers with a 24-h UIE < 150 μg/d was significantly lower compared to that of the healthy volunteers with a 24-h UIE ≥ 150 μg/d (*P* < 0.01; **Table 1**). The healthy volunteers with a 24-h UIE < 150 μg/d had lower saliva values than healthy volunteers with a 24-h UIE ≥ 150 μg/d.

**TABLE 1:** The 24-h UIE of 20 healthy volunteers (group 1) in relation to the self-reported daily iodine intake and SIC, SI/SP, and SI/SU^1^

*n*	24-h UIE,^2^ μg/d	Iodine intake, μg/d	SIC,^3^ μg/L	SI/SP,^3^ μg/g	SI/SU,^3^ μg/mmol
8	<150	126 (80.3–137)^4^	53.8 (32.3–109)^5^	199 (140–260)^5^	14.5 (10.9–17.0)
12	≥150	227 (186–339)	123 (86.1–161)	421 (236–452)	25.0 (19.4–31.9)

1 Data are expressed as medians (IQRs). Abbreviations: 24-h UIE, urine iodine excretion per 24 h; SIC, saliva iodine concentration; SI/SP, salivary-iodine-to-protein-ratio; SI/SU, salivary-iodine-to-urea ratio.

2 The 24-h UIE values <150 and >150 μg/d were defined insufficient and sufficient, respectively.

3 The individual means of saliva sample numbers 2–9 of SIC, SI/SP, and SI/SP were used.

4 Significant at a *P* value < 0.01 using the Mann-Whitney U test.

5 Significant at a *P* value < 0.05 using the Mann-Whitney U test.

### Comparison of salivary iodine and urinary iodine

Median concentrations of iodine in urine and saliva in healthy volunteers, DTC patients, and patients on amiodarone are shown in **Table 2**. The urinary and salivary iodine concentrations, with or without salivary protein or urea correction, were significantly different between the participant groups (*P* < 0.01).

**TABLE 2:** Urinary iodine excretion and salivary iodine, corrected for salivary protein or urea secretion, in healthy volunteers (group 1), DTC patients (group 2), and patients on amiodarone (group 3)^1^

	Healthy volunteers (group 1)^2^	DTC patients (group 2)	Patients on amiodarone (group 3)
*n*	20	10	10
24-h UIE,^3^ μg/d	176 (96.1–213)	26.0 (22.0–37.0)^4^	10.0*10^3^ (7.57*10^3^–11.4*10^3^)
UI/Cr,^3^ μg/gCr	102 (87.7–141)	24.5 (15.0–27.5)	9.32*10^3^ (7.86*10^3^–10.7*10^3^)
SIC,^3^ μg/L	136 (86.3–308)^5^	71.5 (29.5–94.5)	14.3*10^3^ (10.6*10^3^–25.6*10^3^)^6^
SI/SP,^3^ μg/g	412 (275–625)^7^	100 (63.3–139)	46.1*10^3^ (31.5*10^3^–74.2*10^3^)^6^
SI/SU,^3^ μg/mmol	28.4 (16.8–38.0)^7^	7.8 (5.2–19.6)	1.98*10^3^ (1.61*10^3^–4.0*10^3^)^6^

1 Data are expressed as medians (IQRs). Abbreviations: 24-h UIE, urine iodine excretion per 24 h; DTC, differentiated thyroid carcinoma; SIC, saliva iodine concentration; SI/SP, salivary-iodine-to-protein-ratio; SI/SU, salivary-iodine-to-urea ratio; UI/Cr, urinary-iodine-to-creatinine ratio.

2 The first morning saliva sample of the healthy volunteers was used.

3 Significant between the 3 groups at a *P* value < 0.01 using the Kruskal-Wallis test.

4 One DTC patient had a total 24-h urine volume <600 mL: hence, *n* = 9.

5 Two samples were missing: hence, *n* = 18.

6 One sample was missing: hence, *n* = 9.

7 Ten samples were missing: hence, *n* = 10.

Strong correlations were found between 24-h UIE and salivary iodine secretion, corrected for salivary protein and urea (**Table 3**). In addition, salivary iodine secretion, with the correction for salivary protein and urea, correlated significantly with the UI/Cr (Table 3).

**TABLE 3:** Spearman's correlation between urinary iodine and salivary iodine, corrected for salivary protein or urea secretion, in healthy volunteers (group 1), DTC patients (group 2), and patients on amiodarone (group 3)^1^

	SIC,^2^ μg/L	SI/SP,^3^ μg/g	SI/SU,^3^ μg/mmol
24-h UIE, μg/d	0.80^4^	0.90^4^	0.86^4^
UI/Cr, μg/gCr	0.85^4^	0.90^4^	0.92^4^

1 Analyses were performed on morning saliva samples of healthy volunteers (group 1), DTC patients (group 2), and patients on amiodarone (group 3). Abbreviations: 24-h UIE, urine iodine excretion per 24 h; DTC, differentiated thyroid carcinoma; SIC, saliva iodine concentration; SI/SP, salivary-iodine-to-protein-ratio; SI/SU, salivary-iodine-to-urea ratio; UI/Cr, urinary-iodine-to-creatinine ratio.

2 There were 4 missing samples: hence, *n* = 36.

3 There were 12 missing samples: hence, *n* = 28.

4 Significant at a *P* value < 0.01 using Spearman's correlation.

## Discussion

This study demonstrates a strong correlation between salivary and urinary iodine in adults, with an even stronger correlation when corrected for salivary protein or urea. Furthermore, the measurement of iodine secretion by the salivary glands is possible over a broad range, so for clinical practice, salivary iodine could be used as a reflection of the total iodine body pool. In addition, the intra- and inter-individual CVs for salivary iodine were the lowest when corrected for salivary protein or urea.

UIE is widely used to assess dietary iodine intake and status, since more than 90% of the ingested iodine is excreted via urine (17–19). Previous studies comparing UIE with saliva reported similar findings as those in our study (7, 20). In patients with dental caries, a significant correlation of 0.75 between SIC and morning spot urine samples was found (20). In healthy school-aged children, a significant correlation was also found between SIC and 24-h UIE (7). We included samples of participants with a much wider range of iodine intake than those in the studies mentioned above. Furthermore, we corrected for the salivary water content. This correction resulted in even better correlations, which seems to make the use of SI/SP or SI/SU more preferable. Even though participants were instructed to collect saliva without chewing, correction of salivary iodine was performed to correct for potential differences in water content due to possible stimulation of the parotid gland (13, 14, 21).

For the clinical applicability of a salivary iodine measurement, a low intra-individual CV is necessary. Correcting for water content using salivary protein and urea correction decreased the intra-individual CVs for SIC from 63.8% to 37.7% and 26.9%, respectively, and the inter-individual CVs from 77.5% to 41.6% and 47.0%, respectively. Guo et al. (16) demonstrated similar salivary intra-individual CVs, as was reported earlier for 24-h UIE by König et al. (17). In addition, the inter-individual CV for SIC was also comparable with that of the study from Guo et al. (16). In clinical practice, the collection of saliva samples is not recommended before breakfast (16). In our study, the intra-individual CV for SIC improved when the first sampling moment was excluded (SIC CV of 39.8%).

The 24-h UIE, considered the reference standard to determine iodine status, is not only time-consuming and inconvenient but also has high rates of inaccurate collection (up to 50%) and incorrect behavior reported (19, 22, 23). The use of salivary iodine as an indicator of the iodine body pool is more practical and cleaner, and hence is the preferred method.

The salivary iodine measurement could have several clinical purposes. First, it could be helpful in DTC patients to determine the iodine status after the LID to evaluate the effect of the diet in preparation for ^131^I therapy. Second, this method would especially be helpful in DTC patients who have a high iodine status: that is, after receiving iodinated contrast or after the use of amiodarone. This high iodine status could delay the administration of ^131^I because there is a lot of uncertainty about the iodine wash-out period of the body (the period at which the iodine body pool decreased sufficiently for ^131^I therapy) after the use of these substances (10, 24–28). This method could quickly provide information about the iodine status to plan the start of the ^131^I therapy optimally. Third, a saliva iodine analysis could also be a valuable tool for epidemiological purposes (e.g., iodine deficiency in pregnant women or children).

Concerning the optimal time of salivary sampling, Guo et al. (16) demonstrated that in children, the highest correlation between SIC and iodine intake was found after dinner (19:00–19:30), and they recommend saliva sampling after 14:00. The ideal sampling time, with a specific protocol (e.g., food intake restriction before collection), should also be established in a larger group of adult subjects with a broad range of iodine body pools. Furthermore, in their population of school-aged children, Guo et al. (7) calculated an optimal cutoff point for assessing iodine deficiency (SIC < 105 μg/L); however, further research is needed to confirm this and also to establish cutoff points for other age groups.

This study was limited by the low sample size. As a result of our low sample size, the ideal sampling time and the optimal cutoff point for iodine deficiency could not be determined. Furthermore, the self-reported nutrition diaries could be vulnerable to underestimation of the dietary intake. Nevertheless, our study had several strengths. Firstly, we measured salivary iodine in several adult participants with different iodine body pools, providing data about a significant relevant measuring range. Secondly, we corrected salivary iodine values for increased salivary water content due to chewing by measuring salivary protein and urea.

To conclude, measurement of salivary iodine can be performed to assess the total iodine body pool, with the recommendation to correct for salivary protein or urea. Further research is needed to establish the optimal sampling time and clinical cutoff value to determine iodine deficiency.

## Supplementary Material

nxab303_Supplemental_File

## Notes

The research leading to these results has received funding from the Junior Scientific Masterclass Groningen (to BLD).

Author disclosures: The authors report no conflicts of interest.

Supplemental Data are available from the “Supplementary data” link in the online posting of the article and from the same link in the online table of contents available at: https://academic.oup.com/jn.

DJT and ANAvdH-S contributed equally to this study.

Abbreviations used: ^131^I, radioactive iodine; DTC, differentiated thyroid carcinoma; LID, low-iodine diet; LoD, limit of detection; SIC, saliva iodine concentration; SI/SP, salivary-iodine-to-protein ratio; SI/SU, salivary-iodine-to-urea ratio; UI/Cr, urinary-iodine-to-creatinine ratio; UIE, urine iodine excretion; UMCG, University Medical Center Groningen.
