# Supplementary material for: Use of Salivary Iodine Concentrations to Estimate the Iodine Status of Adults in Clinical Practice
Source: J Nutr. 2021 Sep 29;151(12):3671–7. doi: 10.1093/jn/nxab303 (PMC8643657; doi:10.1093/jn/nxab303)
Supplement: nxab303_Supplemental_File [file nxab303_supplemental_file.docx]

**Inductively coupled plasma mass spectrometer assay of iodine**

**Principle**

It is well known that if iodine is present as iodide, destruction of the organic matrix using HNO_3_ is not possible because of losses due to volatile iodine or HI formation. At high pH, the oxidation of iodide to iodine is avoided. Therefore, it is standard to prepare samples in alkaline media to prevent the oxidation of I− to I2 or the formation of HI. It is also essential to destroy the organic matrix with saliva samples to reduce the spectral interferences from carbon species and the possibility of cone blockage in the ICP-MS interface. Thus decomposition with a strong alkali such as sodium hydroxide was used to prepare saliva samples for iodine determination.

**Apparatus and reagents**

Iodine was measured using an inductively coupled plasma mass spectrometer (ICP-MS, Thermo scientific iCap-TQ, Breda, the Netherlands). Alle reagents used were certified standards or MS quality. Water was purified using a MilliQ water purification system (Millipore, USA).

**Calibrator stock solution**

The calibrator stock solution of 1000 mg/L iodine was a certified Iodide Standard for ICP-MS (Sigma 41271) in water.

**Quality Control stock solution**

The quality control stock solution of 1000 mg/L iodine was an independent certified Iodide Standard for ICP-MS (Sigma 41271) in water.

**Internal standard stock solution**

The internal standard consisted of mixing 12.5 mL NaOH 4N and 100 µL Antimony ICP standard (1000 mg/L, VWR 1.70302) in 1000 mL of ultrapure water.

**Calibration curve**

Calibration solutions were prepared by preparing concentrations of 0 (blank), 25, 50, 100, 150, 200 and 250 µg/L iodine in water using the Calibrator stock solution.

**Internal Quality Control samples**

Control samples were prepared by preparing concentrations of 0 (blank), 25, 40, 125 and 225 µg iodine in water using the internal standard stock solution.

**External Quality Control samples**

The laboratory participates in the external quality control program of the SKML (www.skml.nl). Monthy, two external QC samples of iodine in plasma are distributed by the SKML and analysed.

**Sample preparation and measurement**

To 100 µL of saliva, calibrator or control sample 2500 µL internal standard solution was added. Samples were vortex mixed and measured.

**Results of carry over**

Six blank samples were spiked with 10 mg/L potassiumiodide, prepared and analysed. After each sample a blank sample was injected to investigate carry over. A maximum carry over of 0.3% was observed. Conclusion: no significant sample carry-over present.

**Results of external quality control samples**

Results for iodine from sept 2020 - june 2021 (N = 20) averaged 99.2 % with an s.d. of 7.2% and varied from 83.0% to 117.1%. Conclusion: the method is reliable and no adsorption of prepared samles to the equipment occurs.
